# Transcatheter Double Valve Replacement to Treat Aortic Stenosis and Severe Tricuspid Regurgitation with 3D Printing Guidance after Mechanical Mitral Valve Replacement

**DOI:** 10.3390/jcdd9090296

**Published:** 2022-09-05

**Authors:** Mengen Zhai, Yu Mao, Yanyan Ma, Yang Liu, Jian Yang

**Affiliations:** Department of Cardiovascular Surgery, Xijing Hospital, Air Force Medical University, Xi’an 710032, China

**Keywords:** tricuspid valve, transcatheter, paravalvular regurgitation, 3-dimensional printing, renal insufficiency

## Abstract

Background: Transcatheter treatments of tricuspid regurgitation (TR) have been emerging as alternatives for high-risk patients. In contrast to the immobilization of the common transcatheter tricuspid device, using a radial force-independent stent valve device at the native tricuspid annular site has several distinct advantages. Case summary: A 76-year-old patient with renal insufficiency who underwent mechanical mitral valve replacement in 2001 and transcatheter aortic valve replacement in 2021 due to severe aortic stenosis presented with chest pain and shortness of breath. Echocardiography suggested that the flow velocities of the mitral mechanical valve and aortic prosthetic valve were both within the normal range, with no significant paravalvular regurgitation; the tricuspid valve exhibited massive regurgitation (VMAX 258 cm/s, PGMAX 27 mmHg). Due to the high surgical risk, we simulated the procedure with a three-dimensional (3D)-printed model and performed transcatheter tricuspid valve replacement using a LuX-Valve (Ningbo Jenscare Biotechnology Co., Ningbo, China). Discussion: We describe transcatheter tricuspid valve replacement using the LuX-Valve and preprocedural guidance with 3D printing. Postprocedural TR was significantly reduced, indicating that 3D printing plays an important role in preprocedural guidance and that the LuX-Valve was safe and practicable for tricuspid valve replacement.

## 1. Introduction

**Figure 1:**
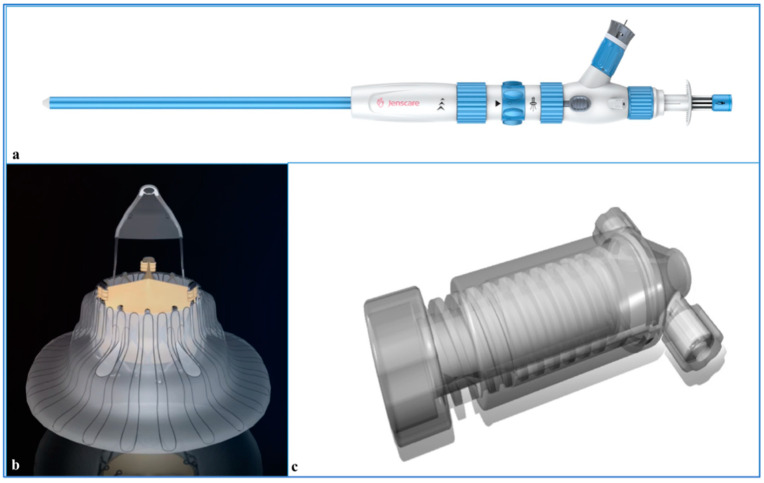
The Lux-Valve is a self-expanding valve. The stent is made of a nickel–titanium alloy, and the biological leaflet is bovine pericardium. The prosthesis is implanted via the right atrial approach and firmly fixed in the tricuspid annulus with its unique anchor device. The large annulus of the prosthesis is located in the right atrium, which also prevents paravalvular leakage. The sheath is 32 Fr, with an adjustable bending function. (**a**) The delivery system of the Lux-Valve; (**b**) right atrial view of the Lux-Valve; (**c**) the loading system of the Lux-Valve.

Tricuspid regurgitation (TR) is a common heart valve disease [1]. Severe TR has been identified as one of the risk factors for long-term survival in addition to age, left ventricular function, or pulmonary artery pressure [2,3]. However, researchers have reported high mortality following traditional cardiac surgery [4]. Transcatheter interventions for TR have been emerging as therapeutic alternatives for high-risk patients [5,6,7,8]. In recent years, a variety of devices have been developed for transcatheter tricuspid valve replacement (TTVR). The LuX-Valve is a novel radial force-independent orthotopic TTVR device (Figure 1). We describe an interesting case of transcatheter double-valve replacement to treat aortic stenosis and severe tricuspid regurgitation in a 76-year-old patient with renal insufficiency after mechanical mitral valve (MV) replacement.

## 2. Case Presentation

**Figure 2:**
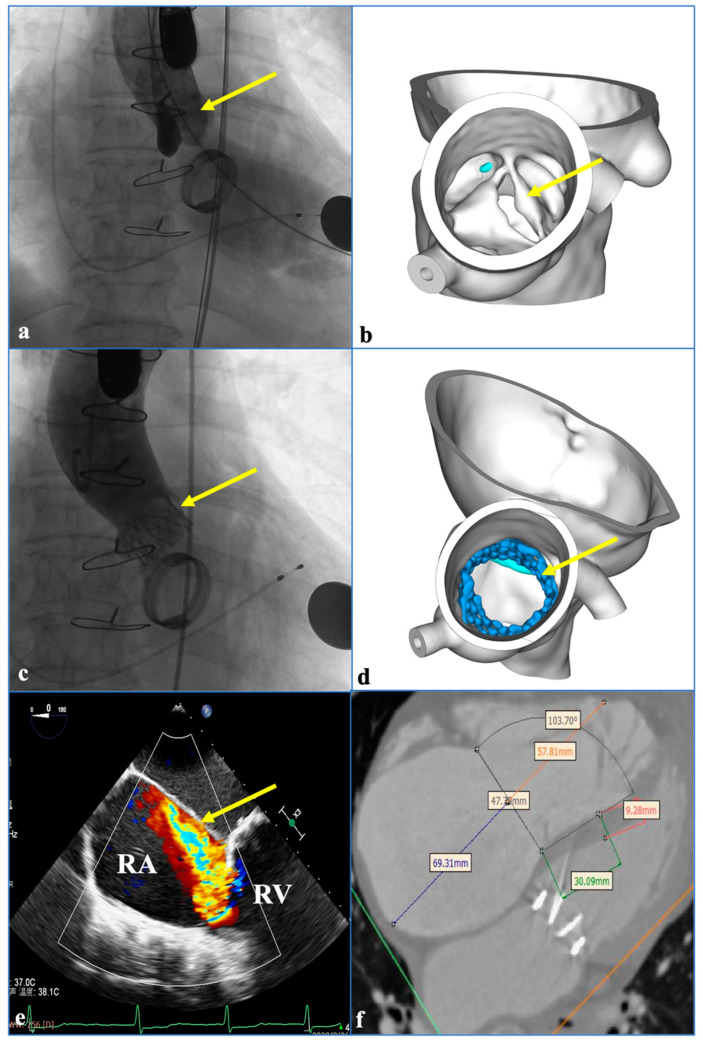
Images before transcatheter tricuspid valve replacement. (**a**) Digital subtraction angiography image before transcatheter aortic valve replacement (TAVR) in 2021; the arrow indicates the patient’s aortic stenosis. (**b**) Three-dimensional reconstruction before TAVR; the arrow points to the patient’s aortic stenosis. (**c**) Digital subtraction angiography shows that the aortic valve stent is fixed securely in place; the arrow points to the stent. (**d**) Three-dimensional reconstruction after TAVR; the arrow points to the stent. (**e**) The yellow arrow points to the massive regurgitation in TV. Chinese words in Figure 2e are temperature. (**f**) The assessment of the computed tomography angiography images before TTVR. RA: right atrium; RV: right ventricle.

A 76-year-old patient underwent mechanical MV replacement in 2001 (Prothesis type: A 29 mm Regent TM MHPJ-505 (St. Jude Medical Inc., Saint Paul, MN, USA)) and transcatheter aortic valve replacement in 2021 (Prothesis type: A 26 mm Venus A (Qiming Medical Co., Ltd., Hangzhou, China)) to treat aortic stenosis (Figure 2a,b). One year later, TR gradually became aggravated. The patient presented with chest pain and shortness of breath. Her lower limbs showed edema. Digital subtraction angiography suggested that the MV and the aortic valve stent were fixed securely in place and moved normally (Figure 2a,d) but that the TV exhibited massive regurgitation in echocardiography images (three bundles; volume—17.9 mL) (Figure 2e). The gap of the TV was approximately 12 mm in systole. After assessing the computed tomography angiography (CTA) scans (Figure 2f), we considered that TTVR might be necessary and suitable for this patient.

## 3. 3-Dimensional Printing and Simulation

**Figure 3:**
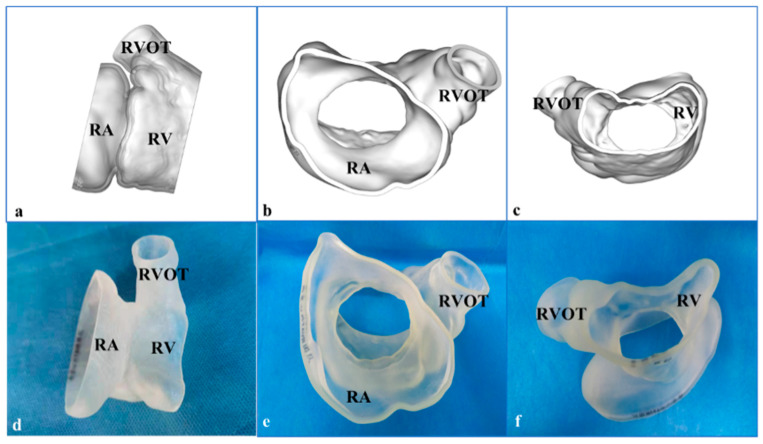
The 3-dimensional (3D)-printed model reconstructed by computed tomography angiography for preprocedural planning and simulation. (**a**) Three-dimensional reconstruction image from the lateral view. (**b**) Three-dimensional reconstruction image from the right atrial (RA) view. (**c**) Three-dimensional reconstruction image from the right ventricular (RV) view. (**d**) The 3D-printed model from the lateral view. (**e**) The model from RA view. (**f**) The model from the RV view. CTA: computed tomography angiography; RA: right atrial; RV: right ventricle; RVOT: right ventricular outflow tract.

We reconstructed the three-dimensional (3D)-printed model from the CTA data (Figure 3a–c). After the image data in Digital Imaging and Communications in Medicine format were collected, Materialise Mimics 21.0 version software (Materialise, Leuven, Belgium) was used to segment the images and export the files into the Standard Tessellation Language (STL) format. Materialise 3-Matic software (Leuven, Belgium) was used for post-processing the STL format files. Finally, STL files were imported into a Polyjet 850 multimaterial full-color 3D printer (Stratasys, Inc., Eden Prairie, MN, USA) (Figure 3d–f). The 3D-printed model was used to simulate the platform. The preoperative 3D-printed model was connected and fixed with the simulator, so as to complete the simulated procedures of TTVR (including right atrium approach, coaxial adjustment of the LuX-Valve delivery system, positioning key and atrial disc expansion, etc.). After the stent released, the morphology, distribution and paravalvular condition were observed clearly. Post-simulation analysis showed a significant reduction in the tricuspid PVL.

## 4. Procedures

**Figure 4:**
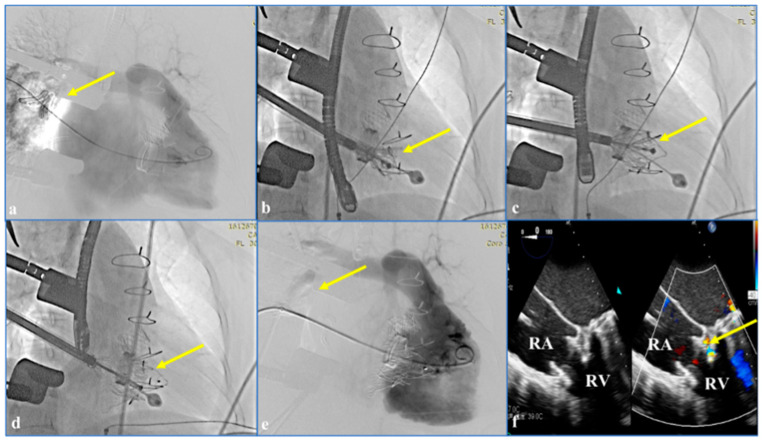
Intra- and postprocedural digital subtraction angiography and transesophageal echocardiography images showing the procedures. (**a**) Computed tomography angiography before the procedure showed massive regurgitation in the tricuspid valve; the arrow indicates the regurgitation. (**b**) The right atrium was incised and the delivery system was delivered into the right ventricle; the arrow points to the delivery system. (**c**) The anchor pin was positioned in the septum; the arrow points to the anchor pin. (**d**) The delivery system was separated from the tricuspid valve and withdrawn from the right atrium; the arrow points to the delivery system. (**e**) Computed tomography angiography shows no significant regurgitation; the arrow points to the tricuspid valve. (**f**) Transesophageal echocardiography displayed only mild TR; the arrow indicates the TR. Chinese words are temperature. RA: right atrium; RV: right ventricle.

CTA scans obtained before the procedure showed massive regurgitation in the TV (Figure 4a). An 8 cm curved incision was made in the right anterior thoracic region. After transesophageal echocardiography (TEE) was used to determine the position of the right atrium, a purse-string suture was used. The center of the purse string stitch was used as the puncture point. A 6 Fr femoral sheath was placed in the right atrium and a 6 Fr pigtail catheter was guided by the guidewire to the pulmonary artery. The pulmonary artery pressure and the right ventricular pressure were 44/20 mmHg and 45/20 mmHg, respectively. The right ventricular CTA showed a large amount of contrast regurgitation into the right atrium, with a right atrial pressure of 47/25 mmHg. Based on the assessment of preprocedural CT and TEE scans, the radial force-independent LuX-Valve was used with a delivery system of 50 mm; the right atrium was incised; and the delivery system was delivered into the right ventricle (Figure 4b). The functional handle was operated to adjust the angle and depth of the TV. The sheath was withdrawn until the valve was fully opened. At the same time, the target position was adjusted using the functional handle so that the anchor pin was positioned in the septum (Figure 4c). The valve was delivered proximally, and the retractor wire of the anchor assembly was then withdrawn. Finally, the delivery system was separated from the valve and withdrawn from the right atrium (Figure 4d). CTA showed that the PVL was significantly reduced (Figure 4e). TEE displayed only mild TR (Figure 4f).

## 5. Follow-Up

**Figure 5:**
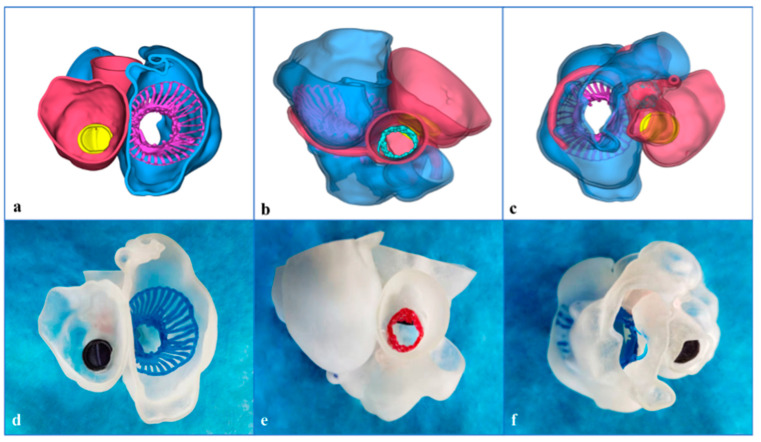
The 3-dimensional (3D)-printed model after the procedure verified the position of the stented valve. In the 3D reconstruction images, the right atrium and right ventricle are blue; the left atrium and the left ventricle are red; the mechanical mitral valve is yellow; the aortic valve stent is green; and the LuX-Valve stent is purple. However, in the 3D-printed model, the mechanical mitral valve is black, the aortic valve stent is red, and the LuX-Valve stent is blue. (**a**) Three-dimensional reconstruction image from the right atrial view; (**b**) 3D reconstruction image from the lateral view; (**c**) 3D reconstruction image from the right ventricular view; (**d**) the 3D-printed model from right atrial view; (**e**) the model from the lateral view; (**f**) the model from right ventricular view.

The patient recovered well and was discharged 5 days after the procedure. At the 30-day follow-up, echocardiography suggested only mild TR, and the electrocardiography images showed the obvious improvement of cardiac function. A 3D-printed model using the postprocedural CTA data verified the position and function of the three valves (Figure 5).

## 6. Discussion

As reported, severe TR is associated with a 5-year survival rate of <50% [9]. Compared with other valves, the TV has a larger annulus, less fibrous tissue in the annulus, and more fragile leaflets [10]. Unfortunately, it remains challenging to find an appropriate approach for TR, particularly for patients who have developed severe right ventricular dysfunction [11,12].

In recent years, there has been a growing interest in TTVR. Several transcatheter therapeutic devices have been designed for TR. Transcatheter therapies of TR have emerged as innovative alternatives of intervention in high-risk patients, showing promising prospects in the early stages [5,8,13]. Boudjemline et al. [14] reported the first percutaneous TTVR device used in animals. Bai et al. [15] reported another preclinical study using similar percutaneous devices in animals. However, neither of these two transcatheter bioprostheses had further clinical applications. Recently, Navia et al. [13] and Asmarats et al. [16] successfully employed the Navigate bioprosthesis device implanted in a degenerated TV ring, showing the feasibility of this device.

In addition, 3D printing, used as a tool to carry out preprocedural planning and training, is critical for preprocedural assessments. Vivid models generated by 3D printers allow interventional cardiologists to better understand specific anatomical structures and simulate interventions. This procedure is especially valuable for high-risk patients. Muraru et al. [17] reported that 3D printing of the TV from 3D TTE data is feasible for the preprocedural guidance of TTVR. Models may help clinicians make diagnoses and make preprocedural plans more accurately and predict in advance possible complications that could occur during the procedure, thereby reducing X-ray exposure time and blood loss.

The specific anatomy of the tricuspid valve makes it difficult to provide a stable support for a traditional radial force-dependent TTVR device. The present study showed that the radial force-independent orthotopic TTVR with the LuX-Valve might be feasible and safe and might achieve acceptable clinical effects in patients with severe TR. Further investigation is needed to evaluate the clinical effect of LuX-Valve in patients with TR.

## 7. Conclusions

We describe TTVR using LuX-Valve and preprocedural guidance with 3D printing. The postprocedural PVL of the TV was significantly reduced, indicating that 3D printing plays an important role in preprocedural guidance. The LuX-Valve was both safe and practicable for TTVR.

## Data Availability

Data were uploaded as suggested by Data Availability Statements in section “MDPI Research Data Policies”.
